# Extended autoantibody panel in Turkish patients with early‐stage systemic sclerosis: Coexpressions and their influences on clinical phenotypes

**DOI:** 10.1002/iid3.1089

**Published:** 2023-12-13

**Authors:** Duygu Temiz Karadağ, Andac Komac, Yesim Erez, Ahmet Merih Birlik, Alper Sari, Ali Akdoğan, Bayram Farisogullari, Gezmiş Kimyon, Emrah Koc, Didem Arslan, Ahmet Karatas, Suleyman Serdar Koca, Nilgün Kasifoglu, Ayten Yazici, Kadir Mutlu Hayran, Ayse Cefle

**Affiliations:** ^1^ Department of Rheumatology Faculty of Medicine, Kocaeli University Kocaeli Turkey; ^2^ Department of Rheumatology Faculty of Medicine, Dokuz Eylül University İzmir Turkey; ^3^ Department of Rheumatology Faculty of Medicine, Hacettepe University Ankara Turkey; ^4^ Department of Rheumatology Faculty of Medicine, Hatay Mustafa Kemal University Hatay Turkey; ^5^ Department of Rheumatology Adana Faculty of Medicine, Cukurova University Adana Turkey; ^6^ Department of Rheumatology Faculty of Medicine, Firat University Elazig Turkey; ^7^ Department of Microbiology Faculty of Medicine, Eskisehir Osmangazi University Eskisehir Turkey; ^8^ Department of Preventive Oncology Faculty of Medicine, Hacettepe University Ankara Turkey

**Keywords:** autoantibody, immunoblot assay, indirect immunofluorescence assay, scleroderma‐specific antibodies, systemic sclerosis

## Abstract

**Background/Aim:**

To investigate the frequency and clinical relevance of an extended autoantibody profile in patients with systemic sclerosis (SSc).

**Materials and Methods:**

In this cross‐sectional study, serum from 100 consecutive patients was subjected to indirect immunofluorescence (IIF) (HEp‐20‐10/primate liver mosaic) and Systemic Sclerosis Profile by EUROIMMUN to evaluate anti‐nuclear antibodies (ANA) and autoantibodies against 13 different autoantibodies in patients with SSc less than 3 years.

**Results:**

Ninety‐three of 100 patients were positive for ANA by IIF. Fifty‐three patients showed single positivity, 26 anti‐topoisomerase antibodies (anti‐Scl70 ab), 16 anticentromere antibodies (ACAs), six anti‐RNA polymerase III antibodies (anti‐RNAPIII ab), one anti‐Ku antibody, one anti‐PM/Scl100 antibody, two anti‐PM/Scl75 antibodies, one anti‐Ro52 antibody, whereas 32 patients had multiple autoantibody positivities. Among classic SSc‐specific autoantibodies, anti‐Scl70 and anti‐RNAPIII abs showed the highest cooccurrence (*n* = 4). One patient was simultaneously positive for anti‐RNAPIII ab and ACA, and one was positive for ACA and anti‐Scl70 ab. The clinical features were not statistically different between single and multiple autoantibody‐positivity for classic SSc‐specific autoantibodies (ACA, anti‐Scl70 ab, and anti‐RNAPIII ab), except for digital ulcer in the multiantibody positive ACA group (*p* = .019).

**Conclusion:**

Based on our results, coexpression of autoantibodies is not uncommon in SSc patients. Although autoantibodies specific to SSc in early disease show generally known clinical features, it remains to be investigated how the coexpression of autoantibodies will affect clinical presentation.

## Graphical abstract

The coexpression of autoantibodies is not uncommon in systemic sclerosis (SSc) patients. Although SSc‐specific autoantibodies generally show known clinical features, the clinical presentation of the coexpression in specific and nonspecific autoantibody positivity continues to be necessary.

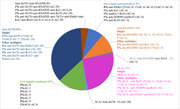

## 1 INTRODUCTION

Systemic sclerosis (SSc) is a rare autoimmune disease characterized by progressive skin and internal organs fibrosis, vasculopathy, and autoantibody production.^1^ Anti‐nuclear antibodies (ANA) can be found in 90%−95% of patients with SSc and SSc‐specific autoantibodies in >80%.^2^ Today, at least 10 SSc‐associated autoantibodies‐anticentromere (ACA), anti‐Scl70 (anti‐topoisomerase I), anti‐RNA polymerase III (anti‐RNAPIII), anti‐U3 ribonucleoprotein (anti‐RNP), anti‐Th/To, anti‐U11/U12 RNP, anti‐PM/Scl, anti‐Ku, anti‐RuvBL1/2, anti‐U1 RNP antibodies (ab)‐ have been reported in SSc patients.^3^, ^4^, ^5^

The clinical course of SSc is not easily predictable because of the clinical and serologic heterogeneity. Nevertheless, from early to established SSc, autoantibodies are used as an indicator in diagnosis, predicting organ involvement, determining prognosis, and making treatment decisions.^6^, ^7^, ^8^ Although recent classification criteria have confirmed their diagnostic utility and have long been used for prognostic stratification of patients, there is still a need to recognize the potential interaction between autoantibodies and their representation on clinical phenotypes of disease.^9^, ^10^, ^11^

The most known and widely used autoantibodies targeting topoisomerase, centromere proteins, and RNA polymerase III have been reported to be mutually exclusive and strongly associated with certain clinical phenotypes.^12^ However, there is also evidence of overlap between these autoantibodies.^3^, ^13^ Although the relationship between the single positivity of some specific autoantibodies and the involvement of certain organs is well known today, the relationship between the positivity of compound autoantibodies and their clinical significance in SSc patients with short disease duration has yet to be investigated in detail.^14^

Therefore, we aimed to investigate autoantibodies' frequency and clinical relevance in the early stage of SSc using an expanded panel of autoantibodies. We also aimed to follow these patients to investigate how the relationship between these autoantibodies and organ involvement progressed. Here we report our initial baseline data.

## 2 METHODS

### 2.1 Patient selection

One hundred patients with SSc were recruited consecutively from six tertiary centers in Turkey, specializing in the care of patients with SSc. We included limited or diffuse cutaneous SSc patients^10^ in the early stage of the disease with a disease duration of <3 years from the first non‐Raynaud symptom,^15^ meeting the 2013 American College of Rheumatology/European League Against Rheumatism (ACR/EULAR) SSc classification criteria.^9^ We included the SSc patients with overlap syndromes, and the overlap syndromes were defined as cases meeting the classification criteria for one or more connective tissue diseases concurrent with SSc. Patients with other comorbidities that could lead to autoantibody positivity were excluded.

Demographic characteristics and clinical and laboratory findings of the patients were recorded. Disease duration was calculated as the time between the onset of the first non‐Raynaud symptom and the enrollment date. Variables included Raynaud's phenomenon, skin and musculoskeletal involvement, pulmonary arterial hypertension (PAH), interstitial lung disease (ILD), renal crisis (ever), gastrointestinal symptoms (dysphagia, reflux, early satiety, constipation, diarrhea) and if recorded any malignancy. The extent of the skin involvement was assessed using the modified Rodnan skin score (mRSS).^16^ Patients with a pulmonary artery pressure above 45 mmHg on echocardiography underwent right heart catheterization because of the strong correlation between this estimated cut‐off level and right heart catheterization.^17^ Pulmonary hypertension was defined as a mean pulmonary arterial pressure of ≥20 mmHg, and precapillary pulmonary hypertension was defined as pulmonary vascular resistance of ≥2 wood units and a pulmonary capillary wedge pressure of ≤15 mmHg on right‐sided heart catheterization.^18^ ILD was defined as the presence of any evidence of pulmonary fibrosis on lung imaging by high‐resolution computed tomography scan. The renal crisis was described as an abrupt onset of severe hypertension (systolic blood pressure [BP] ≥ 180 mmHg and/or diastolic BP ≥ 100 mmHg) without an alternate etiology, with or without microangiopathic anemia or decline in renal function.^19^

This study complied with the Declaration of Helsinki and was approved by the Kocaeli University School of Medicine Ethics Committee, Kocaeli, Turkey, with study number KOU/GOKAEK 2017/347.

### 2.2 Autoantibody analysis

Sera from all patients were tested using a commercially available indirect immunofluorescence (IIF) (ANA, Mosaic Hep‐20‐10/Liver; Euroimmun) assay and line immunoblot assay (Systemic Sclerosis [Nucleoli] Profile EuroLine [IgG]; Euroimmun) simultaneously in a single central laboratory. Serum aliquots were stored at −80°C until the time of testing. The assays were performed according to the manufacturer's instructions. For ANA, results above the dilution of 1:100 were considered positive. The Systemic Sclerosis [Nucleoli] Profile kit contained 13 recombinant antigens: those expressed in *Escherichia coli* (RNA polymerase III [RNAPIII; subunits RP11 and RP155], fibrillarin, the 90‐kd nucleolar protein NOR‐90, and Th/To) or insect cells using the baculovirus system (CENP‐A, CENP‐B, PM/Scl‐100, PM/Scl‐75, Ku, and tripartite motif‐containing protein 21 [TRIM‐ 21]/Ro52) plus platelet‐derived growth factor receptor expressed in mammalian cells and native topo I (Scl70) isolated from calf and rabbit thymus. Sera were analyzed at a dilution of 1:101, and autoantibodies were detected using alkaline phosphatase‐labeled anti‐human IgG. The EuroLine flatbed scanner was used to provide semiquantitative results. Readings obtained with a signal intensity of 0−5, 6−10, 11−25, 26−50, and >50 were defined as negative, borderline, medium, strong, and very strong bands and were given equivalent scores of negative, (+), 1+, 2+, and 3+, respectively. We classified autoantibodies into two groups: classic SSc‐specific autoantibodies‐anti‐Scl70, anti‐ACA and anti‐RNAPIII abs, and SSc‐associated autoantibodies‐all others autoantibodies.

### 2.3 Statistical analysis

Descriptive statistics for clinical and demographic characteristics of the patients are presented as frequency and percentage (%) for categorical variables and mean with standard deviation (mean ± SD) or median with interquartile range (median [Q3−Q1]) according to the distribution of the continuous variables. The distribution normality was assessed visually and through the Shapiro−Wilk test. An independent sample *T*‐test was used to analyze how specific autoantibodies affected clinical outcomes in positive and negative groups and cases of single and multiple positivity. A *χ* ^2^ test was also performed for categorical variables.

In evaluating semiquantitative results, we considered a score of ≥+1 for each autoantibody to be positive. In addition, in statistical analyses, we assumed CENPA and/or CENPB positivity as ACA positive and, similarly, RNAP11 and/or RNA 155 positivity as RNAPIII positive.

Statistical analyses of further demographic and phenotypic data were performed using SPSS, version 20.0 (IBM Inc.). Two‐sided *p* values less than 0.05 were considered statistically significant (*p* < .05).

## 3 RESULTS

### 3.1 Characteristics of the study population and frequency of autoantibodies

Demographic, clinical, and serologic characteristics of the 100 SSc patients are presented in Table 1. Most patients had lcSSc (63%), whereas 36 had diffuse involvement, and only one had sine scleroderma. Ninety‐three out of 100 patients were positive for ANA by IIF. Anti‐topoisomerase antibodies (Anti‐Scl70 ab) was the most frequent (41%) autoantibody, followed by ACA with a rate of 27%. The anti‐RNAPIII ab positivity was 15%. Except for classic SSc‐specific autoantibodies (ACA, anti‐Scl70, or anti‐RNAPIII), anti‐Ro52 was the most common SSc‐associated autoantibody (22%). None of the patients were positive for either anti‐fibrillarin or anti‐PDGF abs.

**Table 1:** Demographic, clinical, and laboratory characteristics of the SSc patients.

	*N* (%) or mean ± SD	
Female	87	(87%)
Age (years)	48.9	±12.2
Disease duration (years)	2.1	±1.4
mRSS	10.8	±10.4
Disease classification		
Diffuse	36	(36%)
Limited	63	(63%)
Sine scleroderma	1	(1%)
PAH	3	(3%)
ILD	33	(33%)
GIS	60	(60%)
DU	14	(14%)
SRC	0	
Myositis	0	
Malignancy	3	(3%)
Overlap	23	(23%)
Anti‐nuclear antibody profile	93	(93%)
ANA patterns		
Speckled	65	(65%)
Nucleolar	13	(13%)
Centromere	29	(29%)
Homogeneous	3	(3%)
Reticular	3	(3%)
Prevalence of autoantibodies		
Anti‐Scl70	41	(41%)
ACA	27	(27%)
CENPA	27	(27%)
CENPB	26	(26%)
Anti‐RNAPIII	15	(15%)
Anti‐RNAP11	9	(9%)
Anti‐RNAP155	13	(13%)
Anti‐NOR90	2	(2%)
Anti‐Th/To	1	(1%)
Anti‐PM/Scl75	8	(8%)
Anti‐PM/Scl100	5	(5%)
Anti‐Ku	6	(6%)
Anti‐Ro52	22	(22%)
Anti‐fibrillarin	0	
Anti‐anti‐PDGF	0	

Abbreviations: ACA, anticentromere; ANA, antinuclear antibody; anti‐RNAPIII, anti‐RNA polymerase III; DU, digital ulcer; GIS, gastrointestinal system; ILD, interstitial lung disease; mRSS, modified Rodnan skin score; PAH, pulmonary arterial hypertension; SRC, scleroderma renal crisis; SSc, systemic sclerosis.

**Figure 1:**
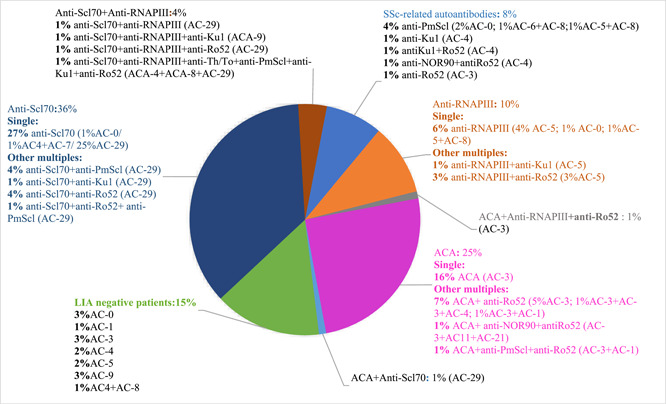
Distribution of the autoantibody positivities of the patients with emphasis on the SSc‐specific autoantibodies (for explanation of AC patterns, please visit https://www.anapatterns.org/nuclear_patterns.php ). AC, anti‐cell; ACA, anti‐centromere antibody; anti‐RNAPIII, anti‐RNA polymerase III; LIA, line immuno‐assay; SSc, systemic sclerosis.

The majority of the patients exhibited single positivity for analyzed autoantibodies. Of 100 patients, 53 were single positivity for any autoantibodies and 48 of which were classic SSc‐specific autoantibodies (26 with anti‐Scl70 ab, 16 with ACA, 6 with anti‐RNAPIII ab), and 5 were SSc‐associated autoantibodies (1 with anti‐Ku ab, 1 with anti‐PM/Scl100 ab, 2 with anti‐PM/Scl75 ab, and 1 with anti‐Ro52 ab). The distribution, copositivity of autoantibodies, and the IFA patterns determined by the International Consensus on Autoantibody Patterns (ICAP: anapatterns.org) are shown in Figure 1 with an emphasis on the SSc‐specific autoantibodies.

There were 32 patients with multiple antibody positivity. Amongst the classic SSc‐specific autoantibodies, anti‐Scl70 and anti‐RNAPIII antibodies showed the highest cooccurrence and were simultaneously positive in 4 patients. One patient was positive for anti‐RNAPIII ab and ACA, and another for ACA and anti‐Scl70 ab simultaneously. All patients with anti‐Ro52 ab except one were also positive for the other antibodies. Eight patients (8%) were positive for SSc‐associated autoantibodies with single or multiple staining. No specific autoantibodies were detected in 15 patients (15%), and ANA was positive in 11 of them.

### 3.2 Associations of autoantibodies with clinical features

The association between the classic SSc‐specific autoantibodies (ACA, Scl70, and RNAPIII) and clinical features was evaluated by comparing the antibody‐positive patients (regardless of being single or multiple positive) with the rest of the study population (Table 2). Overlap syndrome was more common in patients with ACA, and only 1 (3.7%) patient with ACA had ILD. Anti‐RNAPIII ab was associated with a common disease subtype, ILD, and the highest mRSS among the three groups. Among all SSc patients, two out of 3 patients with malignancy were anti‐RNAP ab positive.

**Table 2:** The association of the classic SSc‐specific autoantibodies and clinical features.

	Anti‐Scl70			ACA			Anti‐RNAPIII		
	Positive	Negative	*p*	Positive	Negative	*p*	Positive	Negative	*p*
	*n* = 41	*n* = 59		*n* = 27	*n* = 73		*n* = 15	*n* = 85	
lcSSc	15 (36.6%)	48 (81.4%)	<0.001	27 (100%)	36 (49.3%)	<0.001	5 (33.3%)	58 (68.2%)	0.027
Female	38 (92.7%)	49 (83.1%)	0.229	26 (96.3)	61 (83.6%)	0.177	11 (73.3%)	76 (89.4%)	0.103
ILD	24 (58.5)	9 (15.3%)	<0.001	1 (3.7%)	32 (43.8%)	<0.001	9 (60%)	24 (28.2%)	0.016
PAH	1 (2.4%)	2 (3.4%)	1.000	0	3 (4.1%)	0.561	2 (13.3%)	1 (1.2%)	0.058
Malignancy	0	3 (5.1%)	0.267	0	3 (4.1%)	0.561	2 (13.3%)	1 (1.2%)	0.058
DU	4 (9.8%)	9 (15.3%)	0.551	4 (14.8%)	9 (12.3%)	0.743	2 (13.3%)	11 (12.9%)	1.000
GIS	24 (58.5%)	36 (61%)	0.803	15 (55.6%)	45 (61.6%)	0.581	11 (73.3%)	49 (57.6%)	0.392
Overlap	5 (12.2%)	19 (32.2%)	0.021	11 (40.7)	13 (17.8%)	0.017	1 (6.7%)	23 (27.1%)	0.110
Age	46.9 ± 12.7	51 ± 11.8	0.210	50.4 ± 10.3	48.4 ± 12.9	0.602	55.3 ± 8.5	47.7 ± 12.5	0.026
Disease duration	2.03 ± 1.5	2.14 ± 1.3	0.471	2 ± 1.4	2.13 ± 1.4	0.770	2.6 ± 1.6	2 ± 1.3	0.162
mRSS	14.3 ± 11.5	8.6 ± 9	0.002	6.5 ± 5.2	12.5 ± 11.4	0.024	20.4 ± 13.8	9.2 ± 8.6	0.001

Abbreviations: ACA, anticentromere antibody; anti‐RNAPIII, anti‐RNA polymerase III; DU, digital ulcer; GIS, gastrointestinal system; lcSSc, limited cutaneous systemic sclerosis; ILD, interstitial lung disease; mRSS, modified Rodnan skin score; PAH, pulmonary arterial hypertension.

When we compared the clinical features of the patients in terms of single and multiple ab positivity for each of the classic SSc‐specific autoantibodies (ACA, Scl70, and RNAPIII), the clinical features were not different between the subgroups (Table 3). However, the digital ulcers were more frequent in the multiple ab‐positive ACA group than single positives.

**Table 3:** Clinical features of single and multiple antibody positive patient subgroups within each classic SSc‐specific autoantibody groups.

	Anti‐Scl70 (*N* = 41)			ACA (*N* = 27)			Anti‐RNAPIII (*N* = 15)		
	Single	Multiple	*p*	Single	Multiple	*p*	Single	Multiple	*p*
	*n* = 28	*n* = 13		*n* = 16	*n* = 11		*n* = 6	*n* = 9	
lcSSc	9 (32.1%)	6 (46.2%)	0.386	16 (100%)	11 (100%)	NA	2 (33.3%)	3 (33.3%)	1.000
Female	27 (96.4%)	11 (84.6%)	0.176	15 (93.8%)	11 (100%)	1.000	4 (66.7%)	7 (77.8)	0.634
ILD	17 (60.7%)	7 (53.8%)	0.678	0	1 (9.1%)	0.407	4 (66.7%)	5 (55.6)	0.667
PAH	1 (3.6%)	0	0.490	0	0	NA	1 (16.7%)	1 (11.1%)	0.756
Malignancy	0	0	NA	0	0	NA	2 (33.3%)	0	0.143
DU	2 (7.1%)	2 (15.1%)	0.579	0	4 (36.4%)	0.019	1 (16.7%)	1 (11.1%)	0.756
GIS	15 (53.6%)	9 (69.2%)	0.499	8 (50%)	7 (63.6%)	0.696	4 (66.7%)	7 (77.8%)	0.634
Overlap	4 (14.3%)	1 (7.7%)	1.000	7 (43.8%)	4 (36.4%)	0.508	0	1 (11.1%)	1.000
Age	47.9 ± 12.9	44.8 ± 12.6	0.792	51.5 ± 10.2	48.9 ± 10.7	1.000	60.2 ± 9.9	52.1 ± 5.9	0.088
Disease duration	2 ± 1.5	2.1 ± 1.5	0.810	1.9 ± 1.1	2.1 ± 1.8	0.809	3 ± 1.3	2.3 ± 1.8	0.328
mRSS	13.9 ± 9.3	15.2 ± 15.5	0.690	6.6 ± 5.4	6.3 ± 5.1	0.942	14.5 ± 12.6	24.3 ± 13.9	0.145

Abbreviations: ACA, anticentromere antibody; anti‐RNAPIII, anti‐RNA polymerase III; DU, digital ulcer; GIS, gastrointestinal system; lcSSc, limited cutaneous systemic sclerosis; ILD, interstitial lung disease; mRSS, modified Rodnan skin score; PAH, pulmonary arterial hypertension.

Clinical features of the patients who were positive only for SSc‐associated autoantibodies are shown in Table 4. All the patients except one with anti‐Ku (dcSSc) and another with anti‐PM/Scl100 (sine scleroderma) had limited lcSSc. Only one patient with anti‐Ro52 had overlap syndrome (Sjogren's syndrome [SS]). None of these patients had either ILD, PAH, or malignancy.

**Table 4:** Clinical features of the patients who were positive only for scleroderma‐associated autoantibodies.

	Staining intensity						Clinical and demographic characteristics										
Patient number	Anti‐NOR90	Anti‐Th/To	Anti‐PM/Scl75	Anti‐PM/Scl100	Anti‐Ku	Anti‐Ro52	Subtype	PAH	Gender	ILD	Malignancy	DU	Overlap	GIS	Age	Disease duration (years)	mRSS
1	Neg	Neg	+	Neg	Neg	Neg	Sine	No	Female	No	No	Yes	No	No	36	2	0
2	Neg	Neg	Neg	+	Neg	Neg	Limited	No	Female	No	No	No	No	Yes	46	2	6
3	Neg	Neg	+++	+++	Neg	Neg	Limited	No	Female	No	No	No	No	Yes	55	4	6
4	Neg	Neg	Neg	Neg	+++	Neg	Diffuse	No	Female	No	No	No	No	Yes	54	2	32
5	Neg	Neg	Neg	Neg	+	++	Limited	No	Female	No	No	No	No	Yes	48	1	6
6	+	Neg	Neg	Neg	Neg	+	Limited	No	Female	No	No	Yes	No	Yes	53	4	2
7	Neg	Neg	Neg	+	Neg	Neg	Limited	No	Female	No	No	No	No	No	31	1	4
8	Neg	Neg	Neg	Neg	Neg	+++	Limited	No	Female	No	No	No	Yes	No	71	2	2

Abbreviations: border, borderline; DU, digital ulcer; GIS, gastrointestinal system; ILD, interstitial lung disease; mRSS, modified Rodnan skin score; Neg, negative; PAH, pulmonary arterial hypertension; +, medium staining; ++, strong staining; +++, very strong staining.

When we compared the anti‐Ro52 positive and negative patients, we found that DUs and ACA positivity were more common in anti‐Ro52 positive patients compared to negative ones (27.3% vs. 9%, *p* = .035 and 45.5% vs. 21.8%, *p* = .027, respectively). Anti‐Ro52 positive patients also showed more NOR90 positivity simultaneously (9.1% vs. 0%, *p* = .047). There was no difference between anti‐Ro52 positive and negative patients regarding ILD, PAH, GIS involvement, disease duration, or other autoantibody positivities.

## 4 DISCUSSION

In this study, we investigated an extended autoantibody profile and its association with the clinical manifestations in a group of patients with early‐stage SSc. Consistent with the general knowledge, classic SSc‐specific autoantibodies (anti‐Scl70 ab, ACA, anti‐RNAPIII ab) were more frequent among all tested autoantibodies and exhibited the expected clinical features. However, our results revealed that a substantial proportion of patients were positive for more than one autoantibody, including classic SSc‐specific autoantibodies known as mutually exclusive. Another result that should be considered is that in patients negative for classic SSc‐specific autoantibodies (8%), an extended test profile showed the presence of another autoantibody.

The prevalence of anti‐Scl70, ACA, and anti‐RNAPIII abs in our patients was 41%, 27%, and 15%, respectively. These results were close and consistent with those previously reported in the literature.^4^, ^20^, ^21^, ^22^ However, the frequency of autoantibodies may vary by ethnicity, and data on SSc‐specific autoantibodies from Turkey are limited to the frequencies of anti‐Scl70 and anti‐centromere abs.^23^ In addition, in only one study, the frequency of anti‐RNAPIII antibodies was reported as 2.2%, which was lower than our results.^24^ The difference in results may be because the previous study was conducted in patients with extensive SSc and long disease duration or because the two studies' antibody analysis methods were different.

Our results revealed that a substantial proportion of patients were positive for more than one autoantibody, including classic SSc‐specific autoantibodies. Although classic SSc‐specific autoantibodies (ACA, topo I, and RNAPIII) are thought to be mutually exclusive and do not change from one to another during the disease, there is evidence that they may occur together.^3^, ^4^, ^25^ With the recent advent of multiplexed immunoassays, the notion that these autoantibodies are mutually exclusive is slowly disappearing.^26^ Consistent with these views, we detected the coexpression of classic SSc‐specific autoantibodies in some patients: anti‐Scl70 ab and anti‐RNAPIII Ab positivity in 4, ACA and anti‐RNAPIII ab in 1, and anti‐Scl70 ab and ACA in 1.^27^, ^28^ Regardless of being single or multiple positive, comparison of classic SSc‐specific autoantibody‐positive patients with the rest of the study population showed expected clinical associations with these abs (ACA with IcSSc, anti‐Scl ab with ILD, and anti‐RNAPIII ab with dcSSc and malignancy, etc.). When we compared the single and multiple positivities for each of classic SSc‐specific autoantibodies in terms of clinical involvements, there were no significant differences between subgroups, except for the higher occurrence of DUs in patients who were also positive to ACA. There are uncertainties about the clinical features of multiple antibody‐positive SSc patients in the studies reported to date. Unexpectedly, in 7 dcSSc patients with anti‐RNAPIII ab and ACA, reported by Satoh et al., none had significant organ involvement, such as renal crisis during the disease course.^29^

Similarly, in our study, none of the patients with multiple ab positivity (1 with anti‐Scl70 ab and ACA, and 2 with anti‐RNAPIII ab and ACA positivity) had severe internal organ involvement; interestingly, all had lcSSc. It was difficult to comment on the clinical significance of these multiple autoantibody positivities because the number of patients with SSc‐associated autoantibodies needed to be higher to make detailed comparisons. Although in daily clinical practice, we only evaluate a limited number of autoantibodies in SSc patients, in multiple previous studies, agonistic and classical diagnostic autoantibodies have also been reported. This indicates the importance of poly autoimmunity in the SSc pathogenesis, and a significant percentage of multiple autoantibody positivity in our patients also supports this hypothesis.^30^

Our results showed that, in patients with more than one autoantibody, HEp‐2 IFA specified by the ICAP patterns were consistent with the LIA results to a certain extent. This was especially found for ACA, which is expressed as AC‐3 (centromere), anti‐Scl70 ab as AC‐29 (topo I like), anti‐RNAPIII ab as AC‐5 (nuclear large/coarse speckled), and anti‐PmScl as AC‐8 (homogeneous nucleolar). One of our observations regarding the results of this study was that in patients with multiple autoantibodies, we could not detect all associated patterns. In most situations, HEp‐2 IFA patterns reflect the staining of a single autoantibody in a single cell compartment and conserve basic morphological characteristics that allow their identification according to the ICAP guidelines. However, in daily practice, some situations may not fit this simple rule, and morphological characteristics do not allow the classification as elementary patterns. For example, in the presence of different autoantibodies that react with the same cell compartment, a single pattern may be detected in IFA, and unseen patterns may remain hidden under this pattern.^31^

Anti‐Ro52 was positive in 22% of the patients in our study. Anti‐Ro52 can be found in a variety of autoimmune conditions, including SS, systemic lupus erythematosus, SSc, and inflammatory myositis.^32^ Results from two large cohorts of the German network for systemic Scleroderma and the Canadian Scleroderma Research Group demonstrated that anti‐Ro52 was the second most common autoantibody in patients with SSc.^12^, ^33^ It was our study's third most common autoantibody following anti‐Scl70 and ACA antibodies. Anti‐Ro52‐positive SSc patients were more likely to be older, to have ILD, and to have overlap syndrome compared with anti‐Ro52‐negative patients.^34^ Unlike these results, we found that anti‐Ro52 was more prevalent in ACA‐ab‐positive patients and more associated with DUs. Regarding the aforementioned clinical conditions, it seems essential to re‐evaluate the patients during the follow‐up period of the disease.

Although anti‐RNAPIII ab (15%) positivity was not uncommon in our study patients, none had SRC. In a large SSc cohort of 1325 patients, more than 90% developed SRC within 5 years of SSc onset.^11^ The occurrence estimates of SRC were 6.5%, 7.1%, and 7.6% at 5, 10, and 15 years, respectively. SRC prevalence is lower in North America than in Europe.^35^ SRC is considered less frequent in the Turkish SSc population, with a reported frequency of 3% in a cross‐sectional study from Turkey.^20^ Therefore, the absence of SRC in our study can be partly explained by genetic and geographical differences and the short disease duration of the patients in our study population.

Anti‐PM/Scl antibodies were positive in 10% of the patients in our study. They have been reported to be associated with inflammatory myositis and calcinosis.^36^ In a multinational cohort of SSc, myositis was seen only in subjects positive for both anti‐PM/Scl75 and anti‐PM/Scl100 antibodies.^37^ From this perspective, the three patients with both anti‐PM/Scl75 and anti‐PM/Scl100 antibodies appeared at higher risk for myositis in our study. However, none had myositis or calcinosis at baseline clinical evaluation at enrollment. Despite all these results, continuous monitoring of serum creatine kinase levels in anti‐PM/Scl ab positive patients may be helpful in the further diagnosis of myositis.

One of the limitations of our study was the small sample size. The most important reason for this was to include patients who met the 2013 ACR/EULAR classification criteria and had as short a disease duration. Including only patients with short disease duration might have affected the frequencies of the disease manifestations and prevented reaching statistically significant results in some subgroup analyses, such as associations between disease manifestations and autoantibodies. Another limitation of our study was that the study kits did not contain all SSc‐specific autoantibodies, such as anti‐U1 RNP and anti‐U11/U12 RNP. In addition, the low anti‐Th/To frequency may also be related to the antigen (hPOP1) contained in the kit we used.^38^

In conclusion, our results revealed that among classic SSc‐specific autoantibodies, anti‐Scl70, ACA, and anti‐RNAPIII abs are more common in patients with early SSc, and coexpression of autoantibodies is not infrequent. Testing a broad panel of autoantibodies yielded diagnostic support in 8% of the patients who were negative for classic SSc‐specific autoantibodies. Although autoantibodies specific to SSc in early disease show generally known clinical features, it remains to be investigated how coexpression autoantibodies will affect clinical presentation.

## AUTHOR CONTRIBUTIONS

**Duygu Temiz Karadağ**: Methodology (lead); original draft (lead); formal analysis (lead); conceptualization (equal). **Ali Akdoğan**: Conceptualization (lead); writing—original draft (supporting); writing—review and editing (equal). **Andac Komac, Yesim Erez, Alper Sari, Bayram Farisogullari, Gezmiş Kimyon, Emrah Koc, Ahmet Karatas**: Data curation (equal). **Nilgün Kasifoglu**: Investigation (lead). **Kadir Mutlu Hayran**: Software (lead). **Ahmet Merih Birlik, Didem Arslan, Suleyman Serdar Koca, Ayten Yazici, Ayse Cefle**: Writing—review and editing (equal).

## CONFLICT OF INTEREST STATEMENT

The authors declare no conflict of interest.

## ETHICS STATEMENT

This study was approved by the Kocaeli University School of Medicine Ethics Committee, Kocaeli, Turkey, with study number KOU/GOKAEK 2017/347. Consent was obtained from all participants.

## Data Availability

All data have been included in the manuscript.

## References

[1] Varga J , Trojanowska M , Kuwana M . Pathogenesis of systemic sclerosis: recent insights of molecular and cellular mechanisms and therapeutic opportunities. J Scleroderma Rel Dis. 2017;2(3):137e52.

[2] Stochmal A , Czuwara J , Trojanowska M , Rudnicka L . Anti‐nuclear antibodies in systemic sclerosis: an update. Clin Rev Allergy Immunol. 2020;58(1):40‐51. 30607749 10.1007/s12016-018-8718-8

[3] Patterson KA , Roberts‐Thomson PJ , Lester S , et al. Interpretation of an extended autoantibody profile in a well‐characterized australian systemic sclerosis (scleroderma) cohort using principal components analysis. Arthritis Rheumatol. 2015;67(12):3234‐3244. 10.1002/art.39316 26246178

[4] Mehra S , Walker J , Patterson K , Fritzler MJ . Autoantibodies in systemic sclerosis. Autoimmun Rev. 2013;12(3):340‐354. 22743034 10.1016/j.autrev.2012.05.011

[5] Kayser C , Fritzler MJ . Autoantibodies in systemic sclerosis: unanswered questions. Front Immunol. 2015;6:1‐6. 25926833 10.3389/fimmu.2015.00167 PMC4397862

[6] LeRoy EC , Medsger, Jr. TA . Criteria for the classification of early systemic sclerosis. J Rheumatol. 2001;28:1573‐1576. 11469464

[7] Avouac J , Fransen J , Walker U , et al. Preliminary criteria for the very early diagnosis of systemic sclerosis: results of a Delphi consensus study from EULAR scleroderma trials and research group. Ann Rheum Dis. 2011;70(3):476‐481. 10.1136/ard.2010.136929 21081523

[8] Domsic RT . Scleroderma: the role of serum autoantibodies in defining specific clinical phenotypes and organ system involvement. Curr Opin Rheumatol. 2014;26:646‐652. 25203118 10.1097/BOR.0000000000000113 PMC4299717

[9] Van den Hoogen F , Khanna D , Fransen J , et al. 2013 classification criteria for systemic sclerosis: an American College of Rheumatology/European league against rheumatism collaborative initiative. Ann Rheum Dis. 2013;72:1747‐1755. 24092682 10.1136/annrheumdis-2013-204424

[10] LeRoy EC , Black C , Fleischmajer R , et al. Scleroderma (systemic sclerosis): classification, subsets and pathogenesis. J Rheumatol. 1988;15:202‐205. 3361530

[11] Nihtyanova SI , Sari A , Harvey JC , et al. Using autoantibodies and cutaneous subset to develop outcome‐based disease classification in systemic sclerosis. Arthritis Rheumatol. 2020;72(3):465‐476. 10.1002/art.41153 31682743

[12] Mierau R , Moinzadeh P , Riemekasten G , et al. Frequency of disease‐associated and other nuclear autoantibodies in patients of the German network for systemic scleroderma: correlation with characteristic clinical features. Arthritis Res Ther. 2011;13(5):R172. 22018289 10.1186/ar3495 PMC3308107

[13] Liaskos C , Marou E , Simopoulou T , et al. Disease‐related autoantibody profile in patients with systemic sclerosis. Autoimmunity. 2017;50(7):414‐421. 10.1080/08916934.2017.1357699 28749191

[14] Didier K , Bolko L , Giusti D , et al. Auto‐antibodies associated with connective tissue diseases: what meaning for clinicians? Front Immunol. 2018;9:541. 10.3389/fimmu.2018.00541 29632529 PMC5879136

[15] Medsger Jr. TA. Natural history of systemic sclerosis and the assessment of disease activity, severity, functional status, and psychologic well‐being. Rheumatic Dis Clin North Am. 2003;29(2):255‐273. 10.1016/s0889-857x(03)00023-1 12841294

[16] Clements P , Lachenbruch P , Siebold J , et al. Inter and intraobserver variability of total skin thickness score (modified Rodnan TSS) in systemic sclerosis. J Rheumatol. 1995;22:1281‐1285. 7562759

[17] Hsu VM , Moreyra AE , Wilson AC , et al. Assessment of pulmonary arterial hypertension in patients with systemic sclerosis: comparison of noninvasive tests with results of right‐heart catheterization. J Rheumatol. 2008;35:458‐465. 18203320

[18] Humbert M , Kovacs G , Hoeper MM , et al. 2022 ESC/ERS guidelines for the diagnosis and treatment of pulmonary hypertension. Eur Respir J. 2022;61:2200879. 10.1183/13993003.00879-2022 36028254

[19] Mouthon L , Bussone G , Berezné A , Noël LH , Guillevin L . Scleroderma renal crisis. J Rheumatol. 2014;41(6):1040‐1048. 10.3899/jrheum.131210 24833760

[20] Inanc M , Birlik M , Onat M , et al. The database for systemic sclerosis in Turkey (sys‐watch): clinical features, disease activity, severity and functional impairment of 900 patients. Ann Rheumatic Dis. 2009;68(suppl 3):610.

[21] Meyer O . Prognostic markers for systemic sclerosis. Joint Bone Spine. 2006;73(5):490‐494. 10.1016/j.jbspin.2006.01.022 16798048

[22] Choi MY , Fritzler MJ . Progress in understanding the diagnostic and pathogenic role of autoantibodies associated with systemic sclerosis. Curr Opin Rheumatol. 2016;28(6):586‐594. 10.1097/BOR.0000000000000325 27387266 PMC5029444

[23] Sibanda EN , Dube Y , Chakawa M , Mduluza T , Mutapi F . Systemic sclerosis in Zimbabwe: autoantibody biomarkers, clinical, and laboratory correlates. Front Immunol. 2021;12:679531. 10.3389/fimmu.2021.679531 34858387 PMC8631108

[24] Yayla ME , İlgen U , Düzgün N . An analysis of the relationship between autoantibodies and clinical findings in patients with systemic sclerosis. Turk J Med Sci. 2018;48(1):10‐15. 10.3906/sag-1708-67 29479936

[25] Nandiwada SL , Peterson LK , Mayes MD , et al. Ethnic differences in autoantibody diversity and hierarchy: more clues from a US cohort of patients with systemic sclerosis. J Rheumatol. 2016;43:1816‐1824. 10.3899/jrheum.160106 27481902

[26] Graf SW , Hakendorf P , Lester S , et al. South Australian Scleroderma Register: autoantibodies as predictive biomarkers of phenotype and outcome. Int J Rheumatic Dis. 2012;15:102‐109. 10.1111/j.1756-185X.2011.01688.x 22324953

[27] Tan EM . Anti‐nuclear antibodies in scleroderma. Int J Dermatol. 1981;20:569‐573. 7030985 10.1111/j.1365-4362.1981.tb00835.x

[28] Nakamura RM , Tan EM . Recent advances in laboratory tests and the significance of autoantibodies to nuclear antigens in systemic rheumatic diseases. Clin Lab Med. 1986;6:41‐53. 2420510

[29] Satoh T , Ishikawa O , Ihn H , et al. Clinical usefulness of anti‐RNA polymerase III antibody measurement by enzyme‐linked immunosorbent assay. Rheumatology. 2009;48(12):1570‐1574. 10.1093/rheumatology/kep290 19808694

[30] Miziołek B , Sieńczyk M , Grzywa R , et al. The prevalence and role of functional autoantibodies to angiotensin‐converting‐enzyme‐2 in patients with systemic sclerosis. Autoimmunity. 2021;54(4):181‐186. 10.1080/08916934.2021.1916915 33910447

[31] Cruvinel WM , Andrade LEC , Dellavance A , et al. VI Brazilian consensus guidelines for detection of anti‐cell autoantibodies on HEp‐2 cells. Adv Rheumatol. 2022;62(1):34. 10.1186/s42358-022-00266-z 36071498

[32] Lee AYS . A review of the role and clinical utility of anti‐Ro52/TRIM21 in systemic autoimmunity. Rheumatol Int. 2017;37(8):1323‐1333. 10.1007/s00296-017-3718-1 28417151

[33] Mahler M , Meroni PL , Bossuyt X , Fritzler MJ . Current concepts and future directions for the assessment of autoantibodies to cellular antigens referred to as anti‐nuclear antibodies. J Immunol Res. 2014;2014:1‐18. 10.1155/2014/315179 PMC4020446 24868563

[34] Hudson M , Pope J , Mahler M , et al. Clinical significance of antibodies to Ro52/TRIM21 in systemic sclerosis. Arthritis Res Ther. 2012;14(2):R50. 10.1186/ar3763 22394602 PMC3446416

[35] Hudson M , Ghossein C , Steen V . Scleroderma renal crisis. La Presse Médicale’. 2021;50(1):104063. 10.1016/j.lpm.2021.104063 33548376

[36] Wodkowski M , Hudson M , Proudman S , et al. Clinical correlates of monospecific anti‐PM75 and anti‐PM100 antibodies in a tri‐nation cohort of 1574 systemic sclerosis subjects. Autoimmunity. 2015;48(8):542‐551. 10.3109/08916934.2015.1077231 26334795

[37] Koschik II, RW , Fertig N , Lucas MR , et al. Anti‐PM‐Scl antibody in patients with systemic sclerosis. Clin Exp Rheumatol. 2012;30(2 Suppl 71):12‐16. 22261302

[38] Mahler M , Gascon C , Patel S , et al. Rpp25 is a major target of autoantibodies to the Th/To complex as measured by a novel chemiluminescent assay. Arthritis Res Ther. 2013;15(2):R50. 10.1186/ar4210 23587095 PMC3672760

